# Altered serum microRNAs as biomarkers for the early diagnosis of pulmonary tuberculosis infection

**DOI:** 10.1186/1471-2334-12-384

**Published:** 2012-12-28

**Authors:** Yuhua Qi, Lunbiao Cui, Yiyue Ge, Zhiyang Shi, Kangchen Zhao, Xiling Guo, Dandan Yang, Hao Yu, Lan Cui, Yunfeng Shan, Minghao Zhou, Hua Wang, Zuhong Lu

**Affiliations:** 1 State Key Laboratory of Bioelectronics, School of Biological Science and Medical Engineering, Southeast University, 2 Si Pai Lou, Nanjing, 210096, China; 2 Key Laboratories of Enteric Pathogenic Microbiology, Ministry of Health, Microbiological Laboratory, Jiangsu Center for Disease Prevention and Control (CDC), 172 Jiangsu Rd, Nanjing, 210009, China

**Keywords:** Tuberculosis, MicroRNA, Biomarker, Low-Density Array, qRT-PCR

## Abstract

**Background:**

Pulmonary tuberculosis (TB) is a highly lethal infectious disease and early diagnosis of TB is critical for the control of disease progression. The objective of this study was to profile a panel of serum microRNAs (miRNAs) as potential biomarkers for the early diagnosis of pulmonary TB infection.

**Methods:**

Using TaqMan Low-Density Array (TLDA) analysis followed by quantitative reverse transcriptase polymerase chain reaction (qRT-PCR) validation, expression levels of miRNAs in serum samples from 30 patients with active tuberculosis and 60 patients with *Bordetella pertussis* (BP), varicella-zoster virus (VZV) and enterovirus (EV) were analyzed.

**Results:**

The Low-Density Array data showed that 97 miRNAs were differentially expressed in pulmonary TB patient sera compared with healthy controls (90 up-regulated and 7 down-regulated). Following qRT-PCR confirmation and receiver operational curve (ROC) analysis, three miRNAs (miR-361-5p, miR-889 and miR-576-3p) were shown to distinguish TB infected patients from healthy controls and other microbial infections with moderate sensitivity and specificity (area under curve (AUC) value range, 0.711-0.848). Multiple logistic regression analysis of a combination of these three miRNAs showed an enhanced ability to discriminate between these two groups with an AUC value of 0.863.

**Conclusions:**

Our study suggests that altered levels of serum miRNAs have great potential to serve as non-invasive biomarkers for early detection of pulmonary TB infection.

## Background

Pulmonary tuberculosis (TB) caused by *Mycobacterium tuberculosis* remains a lethal infectious disease and has resulted in increased health care costs, especially in developing countries [1,2]. Early diagnosis of TB infection is essential for the control of the spread of tuberculosis and for adequate antimicrobial therapy against mycobacterial infection. Nevertheless, TB can be a difficult disease to diagnose. The gold standard of TB diagnostics is confirmation with organism growth in selective media, but this culture in clinical specimens requires long incubation time (3–12 weeks) for slow growth of mycobacteria [3]. Sputum smear provides rapid results and is widely used in clinical laboratories, but this conventional method shows low sensitivity. PCR-based nucleic acid amplification assays and Immunological tests brought great progress in TB rapid diagnostics [4-8]. However, endogenous amplification inhibition factor of *M*. *tuberculosis* or unreliable quality control resulting in both false positives and negatives have hampered the clinical use of PCR assays. Immunological tests are time consuming and require confirmation in longitudinal analyses and further functional studies. New biomarkers or methods for TB diagnosis are urgently needed.

Recently, microRNA (miRNA) as a new disease diagnosis biomarker has been intensively studied in many areas, such as various cancers, heart disease, pregnancy, diabetes, psychosis, and various infectious diseases [9-15]. Studies have shown that miR-155 and miR-155* in peripheral blood mononuclear cells (PBMCs) isolated from active TB (ATB) patients exhibited characteristic expression under purified protein derivative (PPD) challenge [16]. MiRNA expression profiles were different in PBMCs from patients with active TB, latent TB infection (LTB), and healthy controls [17]. Differences in miRNA expression of whole-blood between TB and sarcoidosis (SARC) were also detected [18]. Accumulating data have suggested that miRNA could serve as a new potential diagnostic marker for TB infection. Serum miRNAs are present in a stable form that is protected from endogenous RNase activity [19]. Their expression level was consistent among individuals of the same species [19]. Serum, plasma, or other fluid specimens are readily available and noninvasive, these unique characteristics make serum miRNAs become useful biomarkers for disease diagnosis. In this study, we identified the serum miRNAs differentially expressed in patients with active pulmonary TB and explored the potency of serum miRNA expression profiles as an early diagnosis biomarker for TB infection.

## Methods

### Sample collection

A total of 155 participants, including 30 patients with TB infection and 65 healthy subjects were recruited from four districts in the Jiangsu Province between December 2009 and August 2010. Among them, twenty active TB patients and twenty healthy controls were first recruited in the Low-Density Array study. All the participants were recruited for the quantitative RT-PCR assay in validation of the array data. At the time of enrollment, each subject was interviewed and examined clinically and underwent a chest X-ray. Three sputum samples obtained from each TB patient were analyzed using the Ziehl-Neelsen stain and Lowenstein-Jensen culture. Upon admission, all participants were shown to be HIV negative following examination of the presence of HIV antibodies in serum samples using an immunodiagnostic kit for the HIV1/2 antibody (Colloidal Gold; Standard Diagnostics, Korea). Patients were diagnosed on the basis of positive results of sputum smear and TB culture, in combination with clinical symptoms and a chest X-ray examination. Serum samples were collected from patients with active TB (N = 30) who had smear-positive tuberculosis at the time of enrollment and without anti-TB treatment. Healthy controls were recruited at random from people undergoing a regular health check-up. All had normal appearance in chest X-rays and negative results of the IFN-γ release assay (IGRA). Healthy controls were free of TB infection, including active and latent TB infection, and showed no clinical symptoms of any infectious diseases in the routine check-up. As comparisons in array study, 60 serum specimens were collected in parallel from pediatric patients with three other microbial infections: *Bordetella pertussis* (BP), varicella-zoster virus (VZV) and enterovirus (EV). Serum samples were stored at −80°C within 4 h of collection. This project was approved by the Ethics Committee of Jiangsu Provincial Center for Diseases Prevention and Control and written informed consent was obtained from all participants.

### RNA extraction

Five serum pools were created (TB group; control group; three other microbial infection groups (BP, VZV and EV) by combining 20 samples (20 μl per sample) and mixing by inversion and 400 μl of each of these pools was used to extract RNA for assay by TaqMan Low-Density Array (TLDA). A synthetic *Caenorhabditis elegans* miRNA (cel-miR-238 (25 fmol); Takara Biotechnology Co, Dalian, China) was added into each pooled serum as an internal control before starting the isolation procedure. Isolation of total RNA from serum was carried out using mirVana PARIS kits (Ambion, Austin, TX, USA) following the instructions provided by the manufacturer with some modifications. Briefly, the pooled sera were extracted twice with an equal volume of acid-phenol chloroform and RNA was eluted with 100 μl Ambion elution solution according to the instructions provided by the manufacturer. RNA quantity and purity was measured using a NanoDrop spectrophotometer (ND-1000; ThermoScientific, DE, USA). RNA was extracted from individual serum samples (200 μl) used for real-time qRT-PCR assays according to a previously described method [20].

### MiRNA profiling using the TaqMan Low-Density Array

MiRNA profiling assays were performed using the TLDA v2.0 (Applied Biosystems, CA, USA). Each sample was analyzed with an A & B card for duplicate detection of a total of 667 miRNAs together with endogenous and negative controls. Briefly, 50 ng total RNA was added to 4.5 μl reverse transcription (RT) reaction mixture including 0.8 μl Megaplex RT Primer Pools A + B (10×), 0.2 μl dNTPs (100 nM), 1.5 μl MultiScribe Reverse Transcriptase (50 U/μl), 0.8 μl RT Buffer (10×), 0.9 μl MgCl_2_ (25 mM), 0.1 μl RNase inhibitor (20 U/μl) and 0.2 μl nuclease-free water. In order to increase the sensitivity of the TLDA, a pre-amplification was performed after the RT procedure using the TaqMan PreAmp Mastermix and the Megaplex PreAmp Primer Pools A + B (Applied Biosystems). All reactions were carried out according to the protocols recommended by the manufacturer. RT products (2.5 μl) were pre-amplified using the Megaplex PreAmp Primers and reagents. Megaplex RT reactions were diluted 150-fold with water and 450 μl of each diluted product was combined with 450 μl TaqMan 2× Universal PCR Master Mix (No AmpErase UNG) (Applied Biosystems). The sample/master mix for each Megaplex pool (100 μl) was loaded into the array, centrifuged and mechanically sealed with the Applied Biosystems sealer device. qRT-PCR was carried out on an Applied Biosystems 7900HT thermocycler using the cycling conditions recommended by the manufacturer. Real-time PCR data were analyzed using SDS software v2.3 (settings: automatic baseline; threshold, 0.2) and relative miRNA levels were calculated with the RQ Manager v1.2.1 (Applied Biosystems). Serum miRNA levels were normalized against cel-miR-238 (spiked-in synthetic miRNA as an internal control). The threshold cycle (CT) values over 40 were defined as undetectable.

### Candidate miRNA confirmation and quantification by real-time qRT-PCR

Serum sample miRNA was quantified by TaqMan qRT-PCR (Applied Biosystems). Assays were performed using the RT stem-loop primer, PCR primers and probes. RT reactions were performed using the TaqMan miRNA Reverse Transcription Kit and miRNA-specific stem-loop primers in a scaled down (5 μl) RT reaction containing 1.67 μl RNA. The PCRs were carried out with 10 min incubation at 95°C followed by 40 cycles of 95°C for 15 s and 60°C for 1 min in a final volume of 10 μl using a 7900 HT Real-Time PCR System (Applied Biosystems). A typical reaction consisted of 4.5 μl diluted cDNA (1:15), 5 μl TaqMan Universal PCR Master Mix (No AmpErase UNG) and 0.5 μl TaqMan miRNA Assay primer (Applied BioSystems). Each sample was run in triplicate. The CT is defined as the fractional cycle number at which the fluorescence exceeds the defined threshold. The data were analyzed with automatic settings for assigning the baseline. The expression level of miRNA was normalized to miR-16 [21] and was calculated using the ΔΔCT method [22].

### Target gene analysis

Using TargetScan (http://www.targetscan.org), the list of genes predicted to be targeted by the candidated miRNAs were obtained. The predicted target genes were analyzed for different signaling pathways or functions by NCBI DAVID server (http://david.abcc.ncifcrf.gov/tools.jsp) with default setting [23].

### Statistical analysis

For qRT-PCR data, the relative expression levels of each target miRNAs (Log2 relative level) were calculated according to the difference in CT values between the target miRNAs and miR-16 (ΔCT). Statistical analysis was performed with SPSS software version 16.0 (SPSS, Inc., Chicago, USA). A *P*-value <0.05 was considered statistically significant. For each miRNA, a receiver operating characteristic (ROC) curve was generated. The area under curve (AUC) value and 95% confidence intervals (CI) were calculated to determine the specificity and sensitivity of TB infection. To increase the diagnostic accuracy of combined changes in serum miRNA levels, multiple logistic regression analysis was carried out according to previously described methods [24].

## Results

### TB Patient information

The basic demographic characteristics of the participants are showed in Table 1. A total of 65 participants were recruited into this study including 30 TB infected patients (18 men and 12 women; median age, 44.0 ± 14.2 years) and 65 healthy volunteers (35 men and 30 women; median age, 45.3 ± 20.9 years). The 30 active TB patients were diagnosed on the basis of positive sputum smear and TB culture results in combination with clinical symptoms and a chest X-ray examination. There was no significant difference in age and sex distribution between the TB patients and healthy volunteers.

**Table 1:** Demographic characteristics of TB patients and healthy controls

	**Patients group**		**Healthy controls group**	
	**Low**-**Density Array study**	**Validation study**	**Low**-**Density Array study**	**Validation study**
Number of participants	20	30	20	65
Gender (male/female)	10/10	18/12	10/10	35/30
Age (years, mean)	39.2 ± 16.8	44.0 ± 14.2	39.5 ± 16.6	45.3 ± 20.9
Smoking (Yes/no)	4/20	5/30	4/20	10/65
Primary pulmonary lesion by radiography	10	28	0	0
Sputum smear (positive)	20	30	0	0
Culture-positive TB	20	30	NA	NA
BCG vaccination (yes/no)	11/5	18/6	20/0	65/0
IGRA-positive TB	20	30	0/20	0/65
HIV	-	-	-	-

TB, tuberculosis; BCG, Bacillus Calmette-Guérin vaccine; IGRA, IFN-γ release assay. NA, not applicable; HIV, Human immunodeficiency virus.

### MiRNA expression profiling of TB infected serum by TLDA

TaqMan Human miRNA Low-Density Array analysis was performed to identify candidate miRNAs exhibiting altered levels in response to TB infection. Serum miRNAs from TB infected patients were compared with those of healthy controls. Of the 667 miRNAs incorporated in the array, 133 and 166 miRNAs were detected in sera of healthy controls and patients with TB infection, respectively. To identify TB-specific candidate miRNAs, differential expression of miRNAs between patients and the healthy controls were required to meet two criteria: (1) CT values <35 to enable reliable detection, and (2) miRNA levels exhibiting ≥2-fold difference between the patient and control groups [25]. A total of 97 miRNAs met these criteria, 90 of which were up-regulated and seven were down-regulated in TB infected patients compared with healthy controls (See Additional file 1: Table S1 at http://www.biomedcentral.com). Among these, ten miRNAs (miR-210, miR-432, miR-423-5p, miR-134, miR-144*, miR-335, miR-26a, miR-361-5p, miR-889 and miR-576-3p) that were significantly up-regulated (≥5 CT difference between the patient and control groups) were selected for further analysis.

### qRT-PCR analysis of miRNA expression in TB infected serum

**Figure 1:**
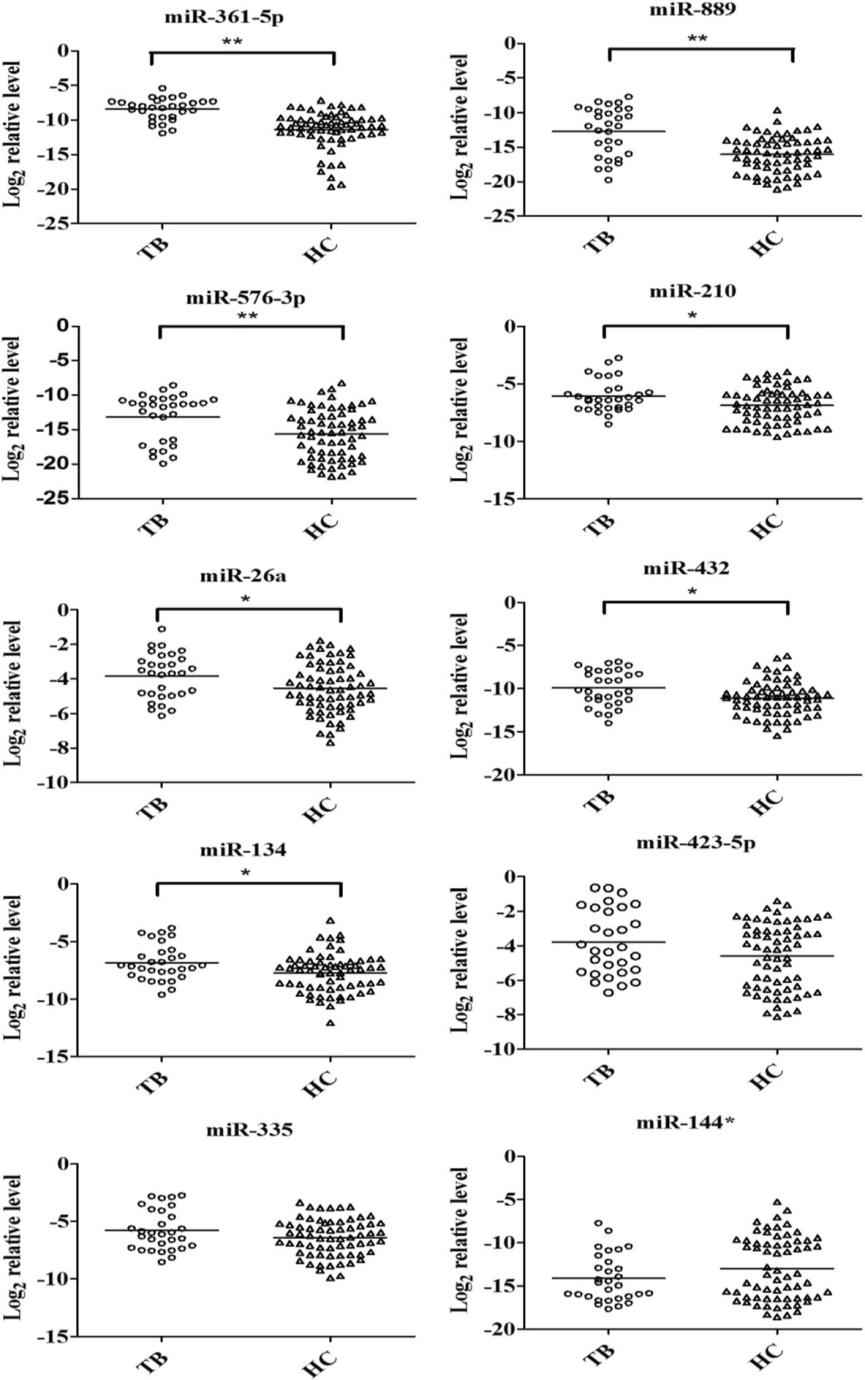
**Ten serum miRNA levels in TB patients and healthy controls were selected for verification using real**-**time qRT**-**PCR in individual TB patients(N** = **30)and healthy controls(N** = **65).** Serum levels of miR-361-5p, miR-889, miR-576-3p, miR-210, miR-26a, miR-432 and miR-134 were significantly higher in TB patients compared with those in the control group (**, *P* < 0.01, * *P* < 0.05) while no significant differences were detected in the expression of miR-423-5p, miR-335 and miR-144*. Expression levels of the miRNAs are normalized to miR-16 (Log_2_ relative level).

The ten candidate miRNAs selected for verification were confirmed and quantified using qRT-PCR (TaqMan miRNA Assays). To date, no reliable endogenous control miRNA has been identified in studying circulating miRNAs, although previous studies have implicated miR-16 as an endogenous control miRNA due to its relatively stable expression level in sera [21,26]. Therefore, miR-16 was used as the endogenous control in this study and the expression levels of candidate miRNAs were normalized to miR-16. The expression levels of miR-361-5p, miR-889, miR-576-3p, miR-210, miR-26a, miR-432, and miR-134 showed significant upregulation in TB infected sera (*P* < 0.05), while no significant differences were detected in the expression of miR-423-5p, miR-335 and miR-144* (*P* > 0.05) (Figure 1).

### Evaluation of the diagnostic potential of miRNAs for TB infection

**Figure 2:**
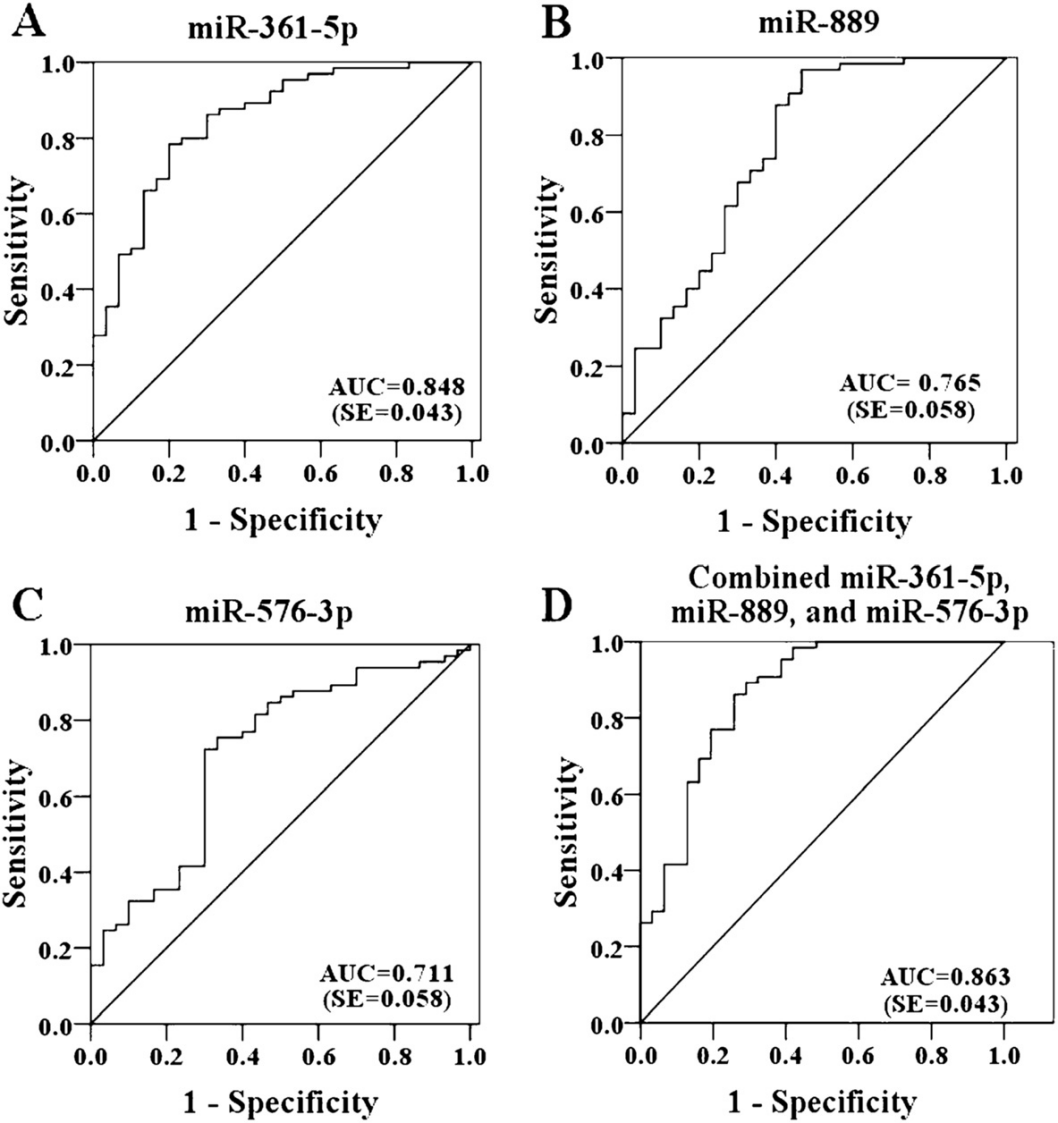
**Receiver operating characteristic** (**ROC**) **curves of differentially expressed miRNAs between TB infected patients and healthy controls.** ROC curves of miR-361-5p (**A**), miR-889 (**B**) and miR-576-3p (**C**) showed a moderate distinguishing efficiency. The combination of miR-361-5p, miR-889 and miR-576-3p showed a slightly higher AUC value of 0.863 (**D**).

**Figure 3:**
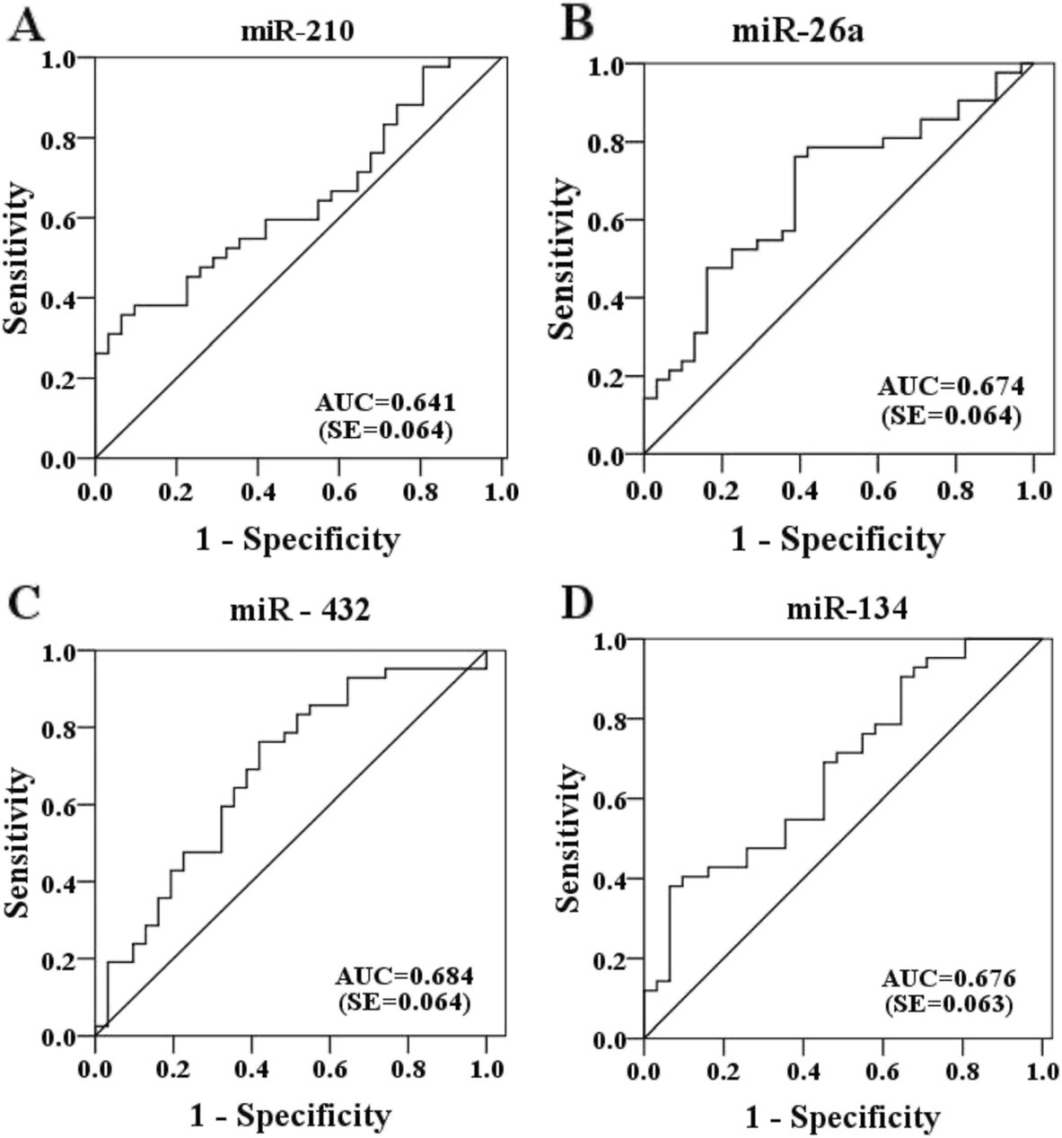
**Receiver operating characteristic** (**ROC**) **curves of differentially expressed miRNAs between TB infected patients and healthy controls.** ROC curves of miR-210 (**A**), miR-26a (**B**), miR-432 (**C**) and miR-134 (**D**) yielded an AUC value less than 0.7.

To investigate the characteristics of these miRNAs as potential diagnostic biomarkers of TB infection, ROC curve analysis was performed on the miRNAs exhibiting significant differential expression. The ROC curves of miR-361-5p, miR-889 and miR-576-3p exhibited a moderate distinguishing efficiency with an AUC value of 0.848 (95% CI, 0.765-0.932), 0.765 (95% CI, 0.652-0.877) and 0.711 (95% CI, 0.597-0.826), respectively (Figure 2A-C). In multiple logistic regression analysis of these three differentially expressed miRNAs, the resulting ROC curve had an AUC value of 0.863 (95% CI 0.778-0.947), which reflects strong separation between the TB infected and control samples (Figure 2D). To verify the specificity of the host miRNAs for TB infection, serum pools from other microbial infection including (BP, VZV and EV) were also detected. The data of three miRNAs (miR-361-5p, miR-889, and miR-576-3p) showed significant difference between TB and three other microbial infection groups (Table 2). MicroR-210, miR-26a, miR-432 and miR-134 showed significant up-regulation in the TB infected group (*P* < 0.05), although the AUC was less than 0.7 (Figure 3), showing poor ability to distinguish the TB infection. In conclusion, the combination of miR-361-5p, miR-889 and miR-576-3p was demonstrated to represent a suitable biomarker that allows efficient differentiation of TB infections from other microbial infections.

**Table 2:** **The ΔΔCt value of three miRNAs in TB and various microbial infections compared with controls measured by TLDA**

**miRNA**	**MT**/**control**	**EV**/**control**	**VZV**/**control**	**BP**/**control**
miR-361-5p	−5.48	0	0	0
miR-889	−5.05	0	−1.47	−1.82
miR-576-3p	−4.51	0	0	0

MT: *Mycobacterium tuberculosis*; EV: enterovirus; VZV: varicella-zoster virus; BP: *Bordetella pertussis.*

### Target gene prediction

To further investigate the possible function of miR-361-5p, miR-889 and miR-576-3p, their predicted target genes were obtained by TargetScan algorithm (239, 509 and 198, respectively). Gene Ontology(GO)analysis showed some genes targeted by miR-361-5p, miR-889 and miR-576-3p involved in immune system development (8, 14 and 8 respectively). For example, among predicted genes (SP1, PGM3, IL10, PIK3R1, RPA1, TGFB2, TGFR1, WEGFA) targeted by miR-361-5p, SP-1 transcription factor (SP1) was a key signaling pathway for IL-10 expression in the lung [27]. Other genes regulated by miR-361-5p and miR-889 are associated with respiratory system development (8 and 18) and lung development (7 and 15).

## Discussion

Circulating miRNAs have been extensively investigated as novel and non-invasive diagnostic and prognostic markers. Many studies have shown that circulating miRNAs serve as potential biomarkers for the early detection of different cancers, such as breast cancer [28], non-small cell lung carcinomas [29,30] and colorectal cancer [31-33]. More recently, the role of miRNA in pathogen-host interactions has attracted attention. Human miRNAs may play important roles in viral replication, limiting antiviral responses, inhibiting apoptosis and stimulating cellular growth [34]. MiRNAs are also associated with immune effects and inflammatory response in bacterial infections [35,36]. However, serum/plasma miRNAs as novel biomarkers for diagnosis of infectious diseases, such as viral or common bacterial infections, remain to be identified. The diagnosis of *M*. *tuberculosis* infection is more difficult to establish than almost every other common bacterial infection [37]. Recently, differential miRNA levels as potential biomarkers in the diagnosis of TB have been described in peripheral blood mononuclear cells (PBMC) [16-18,38] and serum [23] from TB patients. For example, Cheng et al. demonstrated that miR-144* was significantly altered in PBMC from active TB patients [38]. Maertzdorf et al. showed that miR-361-5p, miR-889, and miR-576-3p were under-expressed in TB patients [18]. Our studies demonstrated the potential utility of circulating miRNAs as diagnostic or prognostic biomarkers of TB infection by TLDA analysis of miRNAs that were shown to be differentially expressed in TB patient sera. The majority of these showed up-regulated expression while only seven miRNAs were down-regulated. However, differential expression of miR-144* was not identified by qRT-PCR in our study. This discrepancy may be due to the analysis of different sample types (PBMC and serum). However, serum is more easily obtained and is regarded as more stable and therefore represents a preferred sample type for the analysis of miRNA expression as a circulating diagnostic biomarker. In comparison with a previously published data [23], we didn’t observe miR-29a showing significantly difference in our qRT-PCR results, which may be attributed to the differences in microarray system, sample size, etc.

Tuberculosis is classified as a granulomatous inflammatory condition. Macrophages, T lymphocytes, B lymphocytes and fibroblasts are among the cells that aggregate to form granulomas, with lymphocytes surrounding the infected macrophages. All of these cells secrete miRNAs into the serum. We hypothesized that serum miRNAs were derived from the release of infected epithelial cells as well as from other cell types, including immune cells. The target gene prediction results also showed the miRNAs might involve in regulation of anti-TB immunity, respiratory system development and lung development. Therefore, analysis of a cluster of *M tuberculosis*-associated miRNAs in sera will notably improve the diagnosis of TB infection although the underlying mechanism requires further investigation.

Real-time qRT-PCR was performed in individual serum samples to further verify the ten candidate miRNAs identified by TLDA. Differential expression of seven miRNAs (miR-361-5p, miR-889, miR-576-3p, miR-210, miR-26a, miR-432, and miR-134) was confirmed among these ten candidate miRNAs. To evaluate the efficiency of these dysregulated miRNAs for diagnosis of TB infections, ROC curves were constructed for each miRNA. MiR-361-5p, miR-889, and miR-576-3p showed good ability to efficiently distinguish TB infections from other microbial infections, with an AUC value greater than 0.7 in diagnosing TB infection. MiR-361-5p showed greater ability to distinguish TB infection with an AUC value of 0.848. In this study, we aimed to increase the diagnosis efficiency of these markers by using a combination of several host miRNAs (miR-361-5p, miR-889, and miR-576-3p). Unfortunately, the efficiency was not significantly improved although the AUC value increased slightly from 0.848 to 0.863. In order to verify the specificity of the host miRNAs for TB infection, three different microbial infection serum pools (BP, VZV and EV) were also analyzed by TLDA. The data of three miRNAs (miR-361-5p, miR-889, and miR-576-3p) showed significant difference between TB and three other microbial infection groups (Table 2). Although these three different microbial infection samples were from children, there was no significant difference of expression of three miRNAs between adults and children healthy controls (data not shown). Therefore, miR-361-5p, miR-889, and miR-576-3p could serve as potential molecular markers for TB infection.

Previous studies demonstrated that miR-361-5p was relatively abundant in bleomycin-induced fibrosis in mouse lungs and that its potential target genes may contribute to the understanding of the molecular mechanisms of lung injury and fibrosis [26]. Our studies showed for the first time that higher levels of miR-361-5p are expressed in TB patient sera compared with healthy controls. It can be speculated that this reflects the lung injury caused by TB infection although the mechanism remains to be elucidated. Some evidence suggests that miR-210 can be used as a circulating biomarker for lung cancer among individuals with CT-detected solitary pulmonary nodules [39] and miR-26a can be used for diagnosis of HBV-related hepatocellular carcinoma [40]. MiR-134 has also been reported as a regulator of cell proliferation, apoptosis and migration involving lung septation [41]. Further studies are required to establish their functions in active TB infection.

Several limitations of our study should be noted. Firstly, serum miRNA demonstrated only moderate capacity to differentiate between TB infected patients and controls. Furthermore, only partially dysregulated miRNAs were evaluated and other miRNA combinations may provide more efficient biomarkers. Secondly, our study represents a preliminary investigation of host responses associated with different forms of TB infection. Biomarkers are required for different situations including protection by vaccination, discrimination of latent and active disease to facilitate rapid diagnosis and assessment of treatment outcome and relapse risk [42,43]. Furthermore, additional investigations conducted in larger numbers of patients and healthy volunteers are required to validate our findings.

## Conclusions

In summary, TLDA assays revealed dysregulation of 97 miRNAs in active TB patient sera. A combination of miR-361-5p, miR-889 and miR-576-3p was identified as a potential non-invasive molecular marker for rapid diagnosis of TB infection. The biological mechanism of the dysregulation in these miRNAs and their therapeutic potential in TB infections require further investigation.

## Competing interests

The authors have declared that there are no conflicts of interest exist.

## Authors’ contributions

YQ, LC, YG, ZS, ZL, HW participated in the design and conceived of the study. YQ, KZ, XG, YS performed the experiments. LC, YG participated the statistical analysis. LC helped to draft the manuscript. DY, MZ, HY help to collect the samples. All authors read and approved the final manuscript.

## Pre-publication history

The pre-publication history for this paper can be accessed here:

http://www.biomedcentral.com/1471-2334/12/384/prepub

## Supplementary Material

Additional file 1 Table S1 Differential expressed miRNAs in TB infected patients compared with controls. Click here for file

## References

[B1] Kumar V Abbas AK Fausto N Mitchell RN Robbins Basic Pathology 2007 8 Saunders Elsevier, Pennsylvania 516 522

[B2] WHO Tuberculosis Fact sheet N°104 2010 World Health Organization, Geneva, Switzerland

[B3] Golyshevskaia VI Korneev AA Chernousova LN New microbiological techniques in diagnosis of tuberculosis Probl. Tuberk 1996 6 22 25 9019760

[B4] Foongladda S Pholwat S Eampokalap B Kiratisin P Sutthent R Multi-probe real-time PCR identification of common Mycobacterium species in blood culture broth J Mol Diagn 2009 11 42 48 10.2353/jmoldx.2009.080081 19095775 PMC2607564

[B5] Cheah ES Malkin J Free RC Lee SM Perera N Woltmann G Patel H Kimmitt PT Smith RJ Rajakumar K Barer MR A two-tube combined Taqman/SYBR Green assay to identify mycobacteria and detect single global lineage-defining polymorphisms in Mycobacterium tuberculosis J Mol Diagn 2010 12 250 256 10.2353/jmoldx.2010.090030 20093392 PMC2871733

[B6] Mansoor N Scriba TJ de Kock M Tameris M Abel B Keyser A Little F Soares A Gelderbloem S Mlenjeni S Denation L Hawkridge A Boom WH Kaplan G Hussey GD Hanekom WA HIV - 1 infection in infants severely impairs the immune response induced by Bacille Calmette – Guerin vaccine J Infect Dis 2009 199 982 990 10.1086/597304 19236280 PMC2815505

[B7] Ramos JM Robledano C Masiá M Belda S Padilla S Rodríguez JC Gutierrez F Contribution of Interferon gamma release assays testing to the diagnosis of latent tuberculosis infection in HIV-infected patients: A comparison of QuantiFERON-TB gold in tube, T-SPOT.TB and tuberculin skin test BMC Infectious Diseases 2012 12 169 10.1186/1471-2334-12-169 22849726 PMC3482589

[B8] Elzi L Steffen I Furrer H Fehr J Cavassini M Hirschel B Hoffmann M Bernasconi E Bassetti S Battegay M Improved sensitivity of an interferon-gamma release assay (T-SPOT.TB™) in combination with tuberculin skin test for the diagnosis of latent tuberculosis in the presence of HIV co-infection BMC Infect Dis 2011 11 319 10.1186/1471-2334-11-319 22085801 PMC3226666

[B9] Lawrie CH Gal S Dunlop HM Lawrie CH Gal S Dunlop HM Pushkaran B Liggins AP Pulford K Banham AH Pezzella F Boultwood J Wainscoat JS Hatton CS Harris AL Detection of elevated levels of tumour-associated microRNAs in serum of patients with diffuse large B-cell lymphoma Br J Haematol 2008 141 672 675 10.1111/j.1365-2141.2008.07077.x 18318758

[B10] Zampetaki A Kiechl S Drozdov I Willeit P Mayr U Prokopi M Mayr A Weger S Oberhollenzer F Bonora E Shah A Willeit J Mayr M Plasma microRNA profiling reveals loss of endothelial miR-126 and other microRNAs in type 2 diabetes Circ Res 2010 107 810 817 10.1161/CIRCRESAHA.110.226357 20651284

[B11] Mouillet JF Chu T Hubel CA Nelson DM Parks WT Sadovsky Y The levels of hypoxia-regulated microRNAs in plasma of pregnant women with fetal growth restriction Placenta 2010 31 781 784 10.1016/j.placenta.2010.07.001 20667590 PMC3204658

[B12] Ji F Yang B Peng X Ding H You H Tien P Circulating microRNAs in hepatitis B virus-infected patients J Viral Hepat 2011 18 e242 251 10.1111/j.1365-2893.2011.01443.x 21692939

[B13] Li S Zhu J Zhang W Chen Y Zhang K Popescu LM Ma X Lau WB Rong R Yu X Wang B Li Y Xiao C Zhang M Wang S Yu L Chen AF Yang X Cai J Signature microRNA expression profile of essential hypertension and its novel link to human cytomegalovirus infection Circulation 2011 124 175 184 10.1161/CIRCULATIONAHA.110.012237 21690488

[B14] Shi W Du J Qi Y Liang G Wang T Li S Xie S Zeshan B Xiao Z Aberrant expression of serum miRNAs in schizophrenia J Psychiatr Res 2012 46 198 204 10.1016/j.jpsychires.2011.09.010 22094284

[B15] Cui L Qi Y Li H Ge Y Zhao K Qi X Guo X Shi Z Zhou M Zhu B Guo Y Li J Stratton CW Tang YW Wang H Serum MicroRNA Expression Profile Distinguishes Enterovirus 71 and Coxsackievirus 16 Infections in Patients with Hand-Foot-and-Mouth Disease PLoS ONE 2011 6 e27071 10.1371/journal.pone.0027071 22087245 PMC3210764

[B16] Wu J Lu C Diao N Zhang S Wang S Wang F Gao Y Chen J Shao L Lu J Zhang X Weng X Wang H Zhang W Huang Y Analysis of microRNA expression profiling identifies miR-155 and miR-155* as potential diagnostic markers for active tuberculosis: a preliminary study Hum Immunol 2012 73 31 37 22037148 10.1016/j.humimm.2011.10.003

[B17] Wang C Yang S Sun G Tang X Lu S Neyrolles O Gao Q Comparative miRNA expression profiles in individuals with latent and active tuberculosis PLoS One 2011 6 e25832 10.1371/journal.pone.0025832 22003408 PMC3189221

[B18] Maertzdorf J Weiner J Mollenkopf HJ Network TB BT Bauer T Prasse A Müller-Quernheim J Kaufmann SH Common patterns and disease-related signatures in tuberculosis and sarcoidosis Proc Natl Acad Sci 2012 109 7853 7858 10.1073/pnas.1121072109 22547807 PMC3356621

[B19] Chen X Ba Y Ma L Cai X Yin Y Wang K Guo J Zhang Y Chen J Guo X Li Q Li X Wang W Zhang Y Wang J Jiang X Xiang Y Xu C Zheng P Zhang J Li R Zhang H Shang X Gong T Ning G Wang J Zen K Zhang J Zhang CY Characterization of microRNAs in serum: a novel class of biomarkers for diagnosis of cancer and other diseases Cell Res 2008 18 997 1006 10.1038/cr.2008.282 18766170

[B20] Chang KH Mestdagh P Vandesompele J Kerin MJ Miller N MicroRNA expression profiling to identify and validate reference genes for relative quantification in colorectal cancer BMC Cancer 2010 10 173 10.1186/1471-2407-10-173 20429937 PMC2873395

[B21] Davoren PA McNeill RE Lowery AJ Kerin MJ Miller N Identification of suitable endogenous control genes for microRNA gene expression analysis in human breast cancer BMC Mol Biol 2008 9 76 10.1186/1471-2199-9-76 18718003 PMC2533012

[B22] Livak KJ Schmittgen TD Analysis of relative gene expression data using real-time quantitative PCR and the 2(−△△C (T)) method Methods 2001 25 402 408 10.1006/meth.2001.1262 11846609

[B23] Fu Y Yi Z Wu X Li J Xu F Circulating microRNAs in patients with active pulmonary tuberculosis J Clin Microbiol 2011 49 4246 4251 10.1128/JCM.05459-11 21998423 PMC3232949

[B24] Redell JB Moore AN Ward NH Hergenroeder GW Dash PK Human traumatic brain injury alters plasma microRNA levels J Neurotrauma 2010 27 2147 2156 10.1089/neu.2010.1481 20883153 PMC6468948

[B25] Chanteux H Guisset AC Pilette C Sibille Y LPS induces IL-10 production by human alveolar macrophages via MAPKinases- and Sp1-dependent mechanisms Respir Res 2007 8 71 10.1186/1465-9921-8-71 17916230 PMC2080632

[B26] Xie T Liang J Guo R Liu N Noble PW Jiang D Comprehensive microRNA analysis in bleomycin-induced pulmonary fibrosis identifies multiple sites of molecular regulation Physiol Genomics 2011 43 479 487 10.1152/physiolgenomics.00222.2010 21266501 PMC3110895

[B27] Wulfken LM Moritz R Ohlmann C Holdenrieder S Jung V Becker F Herrmann E Walgenbach-Brunagel G Ruecker VA Muller SC Ellinger J MicroRNAs in renal cell carcinoma: Diagnostic implications of serum miR-1233 levels PLoS One 2011 6 e25787 10.1371/journal.pone.0025787 21984948 PMC3184173

[B28] Cortez MA Welsh JW Calin GA Circulating microRNAs as noninvasive biomarkers in breast cancer Recent Results Cancer Res 2012 195 151 61 10.1007/978-3-642-28160-0_13 22527502 PMC3855311

[B29] Bianchi F Nicassio F Marzi M Belloni E Dall'olio V Bernard L Pelosi G Maisonneuve P Veronesi G Di Fiore PP A serum circulating miRNA diagnostic test to identify asymptomatic high-risk individuals with early stage lung cancer EMBO Mol Med 2011 3 495 503 10.1002/emmm.201100154 21744498 PMC3377091

[B30] Chen X Hu Z Wang W Ba Y Ma L Zhang C Wang C Ren Z Zhao Y Wu S Zhuang R Zhang Y Hu H Liu C Xu L Wang J Shen H Zhang J Zen K Zhang CY Identification of ten serum microRNAs from a genome-wide serum microRNA expression profile as novel noninvasive biomarkers for nonsmall cell lung cancer diagnosis Int J Cancer 2012 130 1620 1628 10.1002/ijc.26177 21557218

[B31] Ng EK Chong WW Jin H Lam EK Shin VY Yu J Poon TC Ng SS Sung JJ Differential expression of microRNAs in plasma of colorectal cancer patients: a potential marker for colorectal cancer screening Gut 2009 58 1375 1381 10.1136/gut.2008.167817 19201770

[B32] Huang Z Huang D Ni S Peng Z Sheng W Du X Plasma microRNAs are promising novel biomarkers for early detection of colorectal cancer Int J Cancer 2010 127 118 126 10.1002/ijc.25007 19876917

[B33] Chen X Ba Y Ma L Cai X Yin Y Wang K Guo J Zhang Y Chen J Guo X Li Q Li X Wang W Zhang Y Wang J Jiang X Xiang Y Xu C Zheng P Zhang J Li R Zhang H Shang X Gong T Ning G Wang J Zen K Zhang J Zhang CY Characterization of microRNAs in serum: a novel class of biomarkers for diagnosis of cancer and other diseases Cell Res 2008 10 997 1006 18766170 10.1038/cr.2008.282

[B34] Grassmann R Jeang KT The roles of microRNAs in mammalian virus infection Biochim Biophys Acta 2008 1779 706 711 10.1016/j.bbagrm.2008.05.005 18549828 PMC2641032

[B35] Ma F Xu S Liu X Zhang Q Xu X Liu M Hua M Li N Yao H Cao X The microRNA miR-29 controls innate and adaptive immune responses to intracellular bacterial infection by targeting interferon-γ Nat Immunol 2011 12 861 869 21785411 10.1038/ni.2073

[B36] Xiao B Liu Z Li BS Tang B Li W Guo G Shi Y Wang F Wu Y Tong WD Guo H Mao XH Zou QM Induction of microRNA-155 during Helicobacter pylori infection and its negative regulatory role in the inflammatory response J Infect Dis 2009 200 916 925 10.1086/605443 19650740

[B37] Saltini C Chemotherapy and diagnosis of tuberculosis Respir Med 2006 100 2085 2097 10.1016/j.rmed.2006.09.015 17113007

[B38] Liu Y Wang X Jiang J Cao Z Yang B Cheng X Modulation of T cell cytokine production by miR-144* with elevated expression in patients with pulmonary tuberculosis Mol Immunol 2011 48 1084 1090 10.1016/j.molimm.2011.02.001 21367459

[B39] Shen J Liu ZL Todd NW Zhang H Liao JP Yu L Guarnera MA Li RY Cai L Zhan M Jiang F Diagnosis of lung cancer in individuals with solitary pulmonary nodules by plasma microRNA biomarkers BMC Cancer 2011 11 374 10.1186/1471-2407-11-374 21864403 PMC3175224

[B40] Zhou J Yu L Gao X Hu J Wang J Dai Z Wang JF Zhang Z Lu S Huang X Wang Z Qiu S Wang X Yang G Sun H Tang Z Wu Y Zhu H Fan J Plasma MicroRNA panel to diagnose hepatitis B virus-related hepatocellular carcinoma J Clin Oncol 2011 29 4781 4788 10.1200/JCO.2011.38.2697 22105822

[B41] Zhang X Wang H Zhang S Song J Zhang Y Wei X Feng Z MiR-134 functions as a regulator of cell proliferation, apoptosis, and migration involving lung septation In Vitro Cell Dev Biol Anim 2012 Jan 19. [Epub ahead of print] 10.1007/s11626-012-9482-3 22259016

[B42] Wallis RS Pai M Menzies D Doherty TM Walzl G Perkins MD Zumla A Biomarkers and diagnostics for tuberculosis: progress, needs, and translation into practice Lancet 2010 375 1920 1937 10.1016/S0140-6736(10)60359-5 20488517

[B43] Zárate-Bladés CR Silva CL Passos GA The impact of transcriptomics on the fight against tuberculosis: focus on biomarkers, BCG vaccination, and immunotherapy Clin Dev Immunol 2011 19 26 30 10.1155/2011/192630 PMC3010624 21197423

