# Supplementary material for: Altered serum microRNAs as biomarkers for the early diagnosis of pulmonary tuberculosis infection
Source: BMC Infect Dis. 2012 Dec 28;12:384. doi: 10.1186/1471-2334-12-384 (PMC3568404; doi:10.1186/1471-2334-12-384)
Supplement: Additional file 1 Table S1 — Differential expressed miRNAs in TB infected patients compared with controls. [file 1471-2334-12-384-S1.doc]

**Table S1:** **differential expressed miRNAs in TB infected patients compared with controls.**

| Detector | △△CT | 2^-^**^△△^**^CT^ |
| --- | --- | --- |
| hsa-miR-210-4373089 | -10.46 | 1412.63 |
| hsa-miR-432-4373280 | -9.47 | 711.39 |
| hsa-miR-423-5p-4395451 | -8.80 | 444.34 |
| hsa-miR-134-4373299 | -8.63 | 396.99 |
| hsa-miR-144*-4395259 | -8.38 | 333.44 |
| hsa-miR-335-4373045 | -7.85 | 230.56 |
| hsa-miR-26a-4395166 | -7.72 | 210.39 |
| hsa-miR-130b-4373144 | -7.67 | 203.73 |
| hsa-miR-26b-4395167 | -7.61 | 194.83 |
| hsa-miR-139-3p-4395424 | -7.60 | 193.58 |
| hsa-miR-29a-4395223 | -7.55 | 187.49 |
| hsa-miR-376c-4395233 | -7.55 | 187.42 |
| hsa-miR-376a-4373026 | -7.53 | 184.80 |
| hsa-miR-425*-4395413 | -7.52 | 183.24 |
| hsa-miR-636-4395199 | -7.26 | 153.29 |
| hsa-miR-299-3p-4373189 | -7.10 | 137.19 |
| hsa-miR-628-5p-4395544 | -6.56 | 94.50 |
| hsa-miR-590-5p-4395176 | -6.54 | 92.94 |
| hsa-miR-185-4395382 | -6.53 | 92.67 |
| hsa-miR-99b*-4395307 | -6.53 | 92.57 |
| hsa-miR-340-4395369 | -6.53 | 92.15 |
| hsa-miR-744-4395435 | -6.50 | 90.21 |
| hsa-miR-409-3p-4395443 | -6.40 | 84.29 |
| hsa-miR-330-3p-4373047 | -6.31 | 79.20 |
| hsa-miR-328-4373049 | -6.14 | 70.69 |
| hsa-miR-433-4373205 | -5.72 | 52.76 |
| hsa-miR-660-4380925 | -5.54 | 46.55 |
| hsa-miR-532-3p-4395466 | -5.53 | 46.33 |
| hsa-miR-505*-4395198 | -5.51 | 45.58 |
| hsa-miR-26a-1*-4395554 | -5.50 | 45.28 |
| hsa-miR-323-3p-4395338 | -5.50 | 45.13 |
| hsa-miR-487b-4378102 | -5.49 | 44.99 |
| hsa-miR-26b*-4395555 | -5.49 | 44.89 |
| hsa-miR-454*-4395185 | -5.49 | 44.83 |
| hsa-miR-598-4395179 | -5.49 | 44.80 |
| hsa-miR-361-5p-4373035 | -5.48 | 44.59 |
| hsa-let-7e-4395517 | -5.48 | 44.50 |
| hsa-miR-886-5p-4395304 | -5.47 | 44.25 |
| hsa-miR-99b-4373007 | -5.44 | 43.49 |
| hsa-miR-409-3p-4395443 | -5.17 | 35.93 |
| hsa-miR-889-4395313 | -5.05 | 33.13 |
| hsa-miR-494-4395476 | -4.97 | 31.32 |
| hsa-miR-200c-4395411 | -4.64 | 25.00 |
| hsa-miR-25-4373071 | -4.55 | 23.38 |
| hsa-miR-196b-4395326 | -4.53 | 23.03 |
| hsa-miR-576-3p-4395462 | -4.51 | 22.78 |
| hsa-miR-127-3p-4373147 | -4.50 | 22.66 |
| hsa-miR-15b-4373122 | -4.42 | 21.39 |
| hsa-miR-410-4378093 | -4.32 | 20.03 |
| hsa-miR-20b-4373263 | -4.27 | 19.35 |
| hsa-miR-199a-3p-4395415 | -4.13 | 17.46 |
| hsa-miR-106a-4395280 | -3.92 | 15.17 |
| hsa-miR-126*-4373269 | -3.80 | 13.90 |
| hsa-miR-93-4373302 | -3.75 | 13.47 |
| hsa-miR-345-4395297 | -3.66 | 12.68 |
| hsa-miR-27a-4373287 | -3.61 | 12.23 |
| hsa-let-7d-4395394 | -3.55 | 11.74 |
| hsa-miR-139-5p-4395400 | -3.54 | 11.67 |
| hsa-let-7g-4395393 | -3.20 | 9.21 |
| hsa-miR-146a-4373132 | -3.12 | 8.67 |
| hsa-miR-21-4373090 | -3.06 | 8.31 |
| hsa-miR-20a-4373286 | -2.90 | 7.46 |
| hsa-miR-24-4373072 | -2.80 | 6.95 |
| hsa-miR-133a-4395357 | -2.78 | 6.87 |
| hsa-miR-301a-4373064 | -2.77 | 6.83 |
| hsa-miR-17-4395419 | -2.62 | 6.14 |
| hsa-miR-320-4395388 | -2.59 | 6.03 |
| hsa-miR-223-4395406 | -2.51 | 5.70 |
| hsa-miR-339-3p-4395295 | -2.37 | 5.16 |
| hsa-miR-103-4373158 | -2.35 | 5.09 |
| hsa-miR-28-3p-4395557 | -2.34 | 5.08 |
| hsa-miR-574-3p-4395460 | -2.33 | 5.01 |
| hsa-miR-92a-4395169 | -2.04 | 4.13 |
| hsa-miR-885-5p-4395407 | -1.99 | 3.99 |
| hsa-miR-193b-4395478 | -1.99 | 3.97 |
| hsa-miR-106b-4373155 | -1.93 | 3.81 |
| hsa-miR-126-4395339 | -1.90 | 3.74 |
| hsa-miR-30d-4373059 | -1.87 | 3.67 |
| hsa-miR-625*-4395543 | -1.81 | 3.50 |
| hsa-miR-145-4395389 | -1.81 | 3.49 |
| hsa-miR-197-4373102 | -1.73 | 3.33 |
| hsa-miR-151-3p-4395365 | -1.62 | 3.08 |
| hsa-miR-30d-4373059 | -1.53 | 2.89 |
| hsa-miR-125a-5p-4395309 | -1.41 | 2.65 |
| hsa-miR-140-5p-4373374 | -1.38 | 2.60 |
| hsa-miR-19a-4373099 | -1.37 | 2.59 |
| hsa-miR-30c-4373060 | -1.32 | 2.50 |
| hsa-miR-425-4380926 | -1.28 | 2.43 |
| hsa-miR-19b-4373098 | -1.05 | 2.07 |
| hsa-miR-142-3p-4373136 | -1.03 | 2.05 |
| hsa-miR-584-4381026 | 6.70 | 0.01 |
| hsa-miR-485-3p-4378095 | 7.51 | 0.01 |
| hsa-miR-768-3p-4395188 | 7.74 | 0.00 |
| hsa-miR-610-4380980 | 8.46 | 0.00 |
| hsa-miR-30b*-4395240 | 8.47 | 0.00 |
| hsa-miR-501-5p-4373226 | 9.48 | 0.00 |
| hsa-miR-571-4381016 | 11.71 | 0.00 |

The different Ct value between two groups was calculated by △△Ct method. The ratio of miRNAs in two groups was calculated by using the equation 2*^−^*^ΔΔCT^.
